# SELF-REPORTED MENTAL AND PHYSICAL HEALTH IN OLDER PATIENTS WITH RHEUMATOID ARTHRITIS

**DOI:** 10.1093/geroni/igad104.2446

**Published:** 2023-12-21

**Authors:** Comfort Anim-Koranteng, Yong Eun, Christfelly Opoku-Agyemang

**Affiliations:** NYCHH-Harlem Hospital Center, Bronx, New York, United States; Harlem Hospital Center, New York City, New York, United States; Harlem Hospital Center, New York City, New York, United States

## Abstract

Rheumatoid arthritis (RA) is the most common autoimmune arthritis with a prevalence of 1% among the American population. RA significantly impacts mortality and is associated with worse quality of life among older adults. Management of RA in older patients is challenging due to comorbidities and frailty. This study compared RA patients’ self-reported mental and physical health between older (65 and over) and younger (18-64) individuals. In the study, we included participants from the All of Us dataset version 6, which includes survey data collected between May 6, 2018, and January 1, 2022, who had one or more diagnosis codes (ICD9/ICD10) of RA and at least one medication exposure for RA. Total of 2904 patients with RA - 1577 older and 1327 younger - were included in the analysis. PROMIS-PH (Patient-Reported Outcomes Measurement System - Physical Health) and MH (Mental Health) score were calculated from the survey result, from 2 to 10: higher score representing better outcome. Average PROMIS-PH score was 6.5 in older patients and 5.9 in younger patients (P< 0.05). Average PROMIS-MH score was 7.2 in older patient and 6.4 in younger patients (P< 0.05). The findings of this study suggest that older patients with RA may have better self-reported mental and physical health than younger individuals. This could be attributed to better disease management, increased resilience, and adaptability. Further research is warranted to understand the underlying causes of the study’s findings.

